# Biopolymer Degradation Analysis: Accelerated Life Testing Study to Characterize Polylactic Acid Durability

**DOI:** 10.3390/ma14195730

**Published:** 2021-09-30

**Authors:** Elias H. Arias-Nava, B. Patrick Sullivan, Delia J. Valles-Rosales

**Affiliations:** 1 Departament of Industrial Engineering and Operations, Instituto Tecnologico Autonomo de Mexico, Río Hondo 1, Altavista, Álvaro Obregón, Ciudad de México 01080, Mexico; 2 Department of Design Production, University of Twente, 7522 NB Enschede, The Netherlands; b.p.sullivan@utwente.nl; 3 Department of Industrial Engineering, New Mexico State University, Las Cruces, NM 88003, USA; dvalles@nmsu.edu

**Keywords:** biomaterial, degradation, accelerated life testing, material testing, PLA

## Abstract

While the degradation of Polylactic Acid (PLA) has been studied for several years, results regarding the mechanism for determining degradation are not completely understood. Through accelerated degradation testing, data can be extrapolated and modeled to test parameters such as temperature, voltage, time, and humidity. Accelerated lifetime testing is used as an alternative to experimentation under normal conditions. The methodology to create this model consisted of fabricating series of ASTM specimens using extrusion and injection molding. These specimens were tested through accelerated degradation; tensile and flexural testing were conducted at different points of time. Nonparametric inference tests for multivariate data are presented. The results indicate that the effect of the independent variable or treatment effect (time) is highly significant. This research intends to provide a better understanding of biopolymer degradation. The findings indicated that the proposed statistical models can be used as a tool for characterization of the material regarding the durability of the biopolymer as an engineering material. Having multiple models, one for each individual accelerating variable, allow deciding which parameter is critical in the characterization of the material.

## 1. Introduction

Polylactic acid (PLA) is a bio-based biodegradable polymer that can be produced from renewable resources including starch from corn and potatoes, as well as from sugars derived from beets, cane, or other agricultural products. Germany, Japan, the Netherlands, and the USA are the principal producers of PLA in the world [1]. With the ability to be easily processed using traditional manufacturing approaches such as injection molding, blow molding, extrusion, and thermoforming; as well as its high strength and thermo-plasticity, PLA can be used in a wide variety of products. Gupta and Kumar [1] found that depending on product use, PLA is well suited and commonly used due to its low molecular weight and reduced degradation time. PLA is considered a well-behaved thermoplastic with a reasonable shelf life for most single use packaging applications and, when disposed of properly, it will hydrolyze to harmless natural products. There are different applications for PLA, as a rigid plastic, biaxially oriented films, or plastic bottles; some examples are meat trays and opaque dairy (yogurt containers), consumer displays and electronics packaging, envelop, display carton windows, short shelf life milk bottles, and bottles used for edible oils [2].

PLA degradation has been studied for several years, however, results regarding the mechanism of degradation are not completely understood yet [3]. Investigation of degradation rates of biopolymers would allow industries and researchers to predict the usable life span possibilities of these type of materials, and learn how to further improve a product usable life. In terms of production costs, PLA presents advantages such as energy usage between 25–55% less than petroleum-based materials; up to this point the challenge is to reduce the PLA manufacturing cost to 1.0 US$/kg; lower energy use in the production is potentially one of the keys concerning overall cost [4].

**Figure 1:**
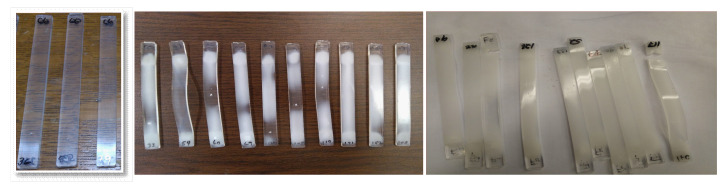
Intersection point in time and 10% of degradation.

The use of PLA products continues to grow, making it increasingly necessary to better understand the accelerated failure process; knowing the degradation rate allows for the prediction of when material failure will occur. This accelerated life testing requires a model representation to understand the parameter changes. A powerful knowledge/understanding tool based on degradation models can be used to determine the predictability of the usable life of PLA materials and products. In general, most polymers experience degradation due to factors such as heat, light, oxygen, and/or water, the effects of these factors can be appreciated in Figure 1. The level of degradation of a polymeric material depends on its ability to absorb UV light (due to the presence of catalyst residues such as hydro peroxide and carbonyl groups) and/or water. Exposure of polymers to UV light irradiation leads to main chain scission causing mechanical deterioration and breaking into small pieces; this consequently allows oxygen and microorganism to degrade the polymer [5].

In this paper, a method to predict and model the degradation of PLA and predict the biopolymer lifetime is presented. Based on past research this model includes multiple accelerating factors including UV light, humidity, and temperature. To support industrial needs the model can adjust according to biopolymer application needs and provide evidence based data for to facilitate decision making during product design and manufacturing phases. The experiment stated three hypotheses:

**Hypothesis** **1.** *UV light, humidity, and temperature exposure have a statistical difference in the degradation rate of PLA after an accelerated weather exposure of 2000 h.*

**Hypothesis** **2.** *The experiment evaluated the premise that UV light exposure has a significant effect on PLA degradation rate after an accelerated weather exposure of 2000 h.*

**Hypothesis** **3.** *Finally, this model evauated a third hypothesis that states: acceleration time has effects on the PLA samples, after 250, 500, 1000, and 2000 h of accelerated condition exposure.*

### Background

Polylactic acid (PLA) has been widely used in many applications for the past decade with some limitations in its durability properties. However, Farah et al. [4] commented that in order to expand the PLA applications in a more competitive market, the mechanical properties, thermal stability, and particularly the degradation rate must be improved. Work has been done in this regard to improve the stiffness at elevated temperatures as well as to reduce the production cost [4]. Particularly, Müller explained the need for improving degradation methods focus on determining the degradation process of the material. Biodegradability test particularly explored the evaluation of low-weight substances and then modified to the environmental condition in which the polymer is being exposed [6]. Previous studies and models related to biopolymers were analyzed and used to compare the final results of this research, in the following Table 1, a brief description of eight modeling approaches are presented.

Previous experiments in degradation analysis were considered as baseline for this research. Garlotta [15] described a complete study of PLA, mechanical properties, analysis in different situation and variables (i.e., molecular weight). PLA degradation depends on several factors, Garlotta [15] studied ways to determine weight molecular changes with different crystallization’s temperature. Hoshino and Isono [16] experimented with several aliphatic polyesters and their degradation behavior (PLA among them). The experimentation was carried out for 100 days, where 2 cm × 2 cm polyester films were placed in an enzyme solution, the samples were weighted at the beginning and at the end of the experiment in order to evaluate the ratio loss as a biodegradability parameter. Iwata [17] experimented with PLA, the polymer was degraded at 55 ${}^{\circ }$C in a pH 8.5 solution for 20 days. Degradation was documented in three stages: (1) film was rough, (2) film was visibly broken down, (3) finally the film disappeared. PLA’ molecular weight was analyzed for 50 days of incubation and it was concluded that degradation resulted from two processes: chemical hydrolysis, where PLA went into oligomers; and enzymatic hydrolysis from the oligomers to monomers. Fukushima [18] studied PLA from l-lactic acid, d-lactic acid and a mixture of both. They synthesized the PLA using a fermentation process with glucose from corn. They studied the degradation mechanism of PLA and its nanocomposites prepared with two montmorillonites at 5% weight ratio. Photodegradation by UV exposure was also explored. It was found that an average of 2000 h of weathering exposure could decrease the modules of elasticity by 12% and no significant change in strength, but with similar exposure in addition with water spray cycle, it decreased flexural and strength respectively from 52 and 34%, [19]. Kaczmarek [20] presented a study about the accelerated photo-degradation of polymers. Photo-degradation depends not only on the polymer’s chemical structure but also on factors such as: defects in chains (molecular); external impurities; physical state and morphology of sample; atmosphere and temperature, among others.

## 2. Materials and Methods

### 2.1. Degradation Testing

Accelerated lifetime (ALT) is a process where the product or material is stressed under accelerated conditions; voltage, temperature, humidity, and UV light to obtain useful results to predict the lifetime of a material/product [21]. Mun et al. combined ALT with destructive characteristics when few or no failures are observed during experimentation under normal conditions [22]. Accelerated test responses could be similar among different models for acceleration, depending on the specific test used during experimentation. The main difference is that using different statistical analysis approaches to the results could lead to a different statistical model. The final goal of any accelerated degradation test when interpreting the results is to be able to extrapolate the information collected during the experimentation, then to process the information and be able to create a particular model. The results of changes in these variables are fit to a statistical model to describe the effect that the experimental variables have on the degradation/failure process [23]. Meeker et al. [12], showed that the information provided and collected from any accelerated degradation test could be analyzed and extrapolated through a physical reasonable statistical model to estimate lifetime or specific time performance under lower, natural or normal conditions. In order to achieve a good general degradation model is important to have models for the individual degradations process, the combination of these is key to the creation of reliable statistical/mathematical models.

### 2.2. Experimentation

The experimentation plan is illustrated in Table 2, where each batch is represented as follows: batch 1 of color factor C*1*, batch 2 of color sample C*2*, and so on; flexural batches represented by (F*n*), and tensile by (T*n*). The experiments were carried out under the guidelines of ASTM’s standards (American Society for Testing and Materials). The experiment included 10 replications on each test/code, the ASTM’s standard recommended at least six specimens to validate the experiment and it was decided to include 10 to minimize the variability and the chances of ending up missing important information. It was expected that PLA would show rapid visual degradation within the first 250 h, and then slow down the degradation rate. This leads to determine intervals of 500 h (500, 1000, 1500, and 2000). The selected times are based on the methodology and previous experiments in the areas of degradation [3].

Poly lactic acid samples were tested for accelerated degradation using the weatherometer Ci5000 Xenon-Arc Weather-Ometer, ATLAS manufacturer, Mount Prospect, IL, USA. This test lasted for 2000 h (around 3 months). The set up parameters for the accelerated weathering conditions were set according to the ASTM D2565-1. Cycle #1 was selected and it is described as follows: 102 min of light exposure only followed by 18 min of light with a water spray (102/18) cycle. Temperature of exposure was 63 ± 2 °C; the irradiance was 0.35 ± 0.02 W/m^2^ at 340 nm. Prior to set the samples in the weatherometer, samples were coded and randomized in order to get more reliable statistical results.

### 2.3. Material Testing

Tensile testing was conducted at different points of time (intervals are shown in Table 1, the machine used was a 5882 Floor Model Testing Systems,100,000 N (22,500 lb.), INSTRON manufacturer, Norwood, MA, USA.This equipment performs tensile and compression testing. Force data were collected as load (given in Newton) applied through time until reaching a fracture point. Equation (1) shows the Ultimate Tensile Strength (UTS) calculation for sample-7 of 250 h using the raw data, for this calculation, the maximum load is used (842.5 N).

(1) $$UTS=\frac{Load(N)}{Cross-sectional\;area({\mathrm{mm}}^{2})}=\frac{842.5256\;N}{\left(3.18\;\mathrm{mm}\;\times \;3.20\;\mathrm{mm}\right)}=82.79\;\mathrm{Mpa}.$$

Flexural testing was performed based on a three-point test. The three-point test involves placing the bar specimen sample between two points and then applying force in the middle to induce the material to bend or break. The machine used was the INSTRON 5882 Floor Model Testing Systems. Maximum stress and strain were calculated on the incremental load applied and it is the one used in the experimentation of this research.

For color testing, the L*a*b* model for colorimetry measure developed by the Commission International d’Eclairage (CIE) was used. CIE established color testing as a standard in a technical report publication 15.2 (1986). Here, color represents the lightness and it is described with two parameters-axis where the L* axis that runs from 100 to zero, 100 represents perfect reflecting diffuser and zero represents the black color. The a* and b* axes are the two chromatic components (ranging from −120 to 120), the a* component goes from green (−120) to red (120). The b* component goes from blue to yellow; blue (−120) and yellow (120). Figure 1, illustrate the visual changes of the samples through time, from left to right samples from times 0, 500, and 2000 h. are shown.

### 2.4. Statistical Analysis

As stated in the Hypothesis 1, one of the things presented in this paper is a study to determine if there is a significant difference in the multivariate analysis of variance. Here, four different tests are presented: Wilks, Pillai, Hotelling–Lawley, and Roy test. All the test results had a *p*-value < 0.005, therefore, the null hypothesis Ho was rejected. This indicated that the multivariate analysis of variance was statistically significant. Then, individual statistical testing for the three responses were conducted in order to identify what variables were significant in the statistical difference of the multivariate analysis. The results indicated that the three of them were significant. The tensile test, the flexural test, and the color test had *p*-values of 1.41 × 10${}^{-6}$, 5.18 × 10${}^{9}$, and 1.52 × 10${}^{-6}$ respectively.

A follow-up analysis was conducted to determine if the data followed a multivariate normal distribution. For this assessment a Skewness and Kurtosis test was conducted, the null hypothesis for this test was that “the sample data are not significantly different than a normal population.” In this case, probabilities > 0.05 indicated that data were coming from a normally distributed population. Probabilities < 0.05 indicated that data were not normally distributed. The results indicated that the effect of the independent time variable or treatment effects was highly significant. At this point, the multivariate test was significant among all three variables.

To test the UV light effect an extra analysis of variance was conducted only between the batch labeled F5 (UV exposure 2000 h) vs. F6 (no UV exposure after 2000 h), see Table 2 experimental plan. The results are presented in table 7.24. With a *p*-value of 0.00000225, the null hypothesis was rejected. Alternatively, a test for nonparametric analysis of variance was presented and the conclusion regarding the null hypothesis stayed the same, rejecting the null hypothesis with a *p*-value of 0.00016. It can be concluded that there was a statistically significant effect of the UV light exposure in Poly lactic acid degradation rate degradation rate PLA after 2000 h. of accelerated weathering exposure.

## 3. Results

The result of the accelerated weathering experimentation allowed for a series of degradation models to be created using two different approaches, the Gaussian and Accelerated destructive degradation test (ADDT). The models are based on each response variable and are based on mathematical approaches as indicated by [24]; mechanical approaches presented by Kruse et al. [7]; stochastics approached stated by Padgett et al. [8], and chemical approached like the one presented by Kostoglou et al. [11]. Gaussian process methodology was used for every response using R software. Gaussian approach uses an algorithm to optimize and find the best fit for data as studied by Cheng et al. [25]. The Gaussian approach states that let Y(t) and L(t) for *t*≥ 0 denote the values for the variable that is analyzed, therefore the model would be expressed in the following Equations (2) and (3). When θ is the random variable representing the unit-to-unit variation and assumed to follow $N(\eta ,\sigma^{2}\eta )$; and β(t) states the Brownian motion ( also known as Wiener process), σβ is representing the diffusion coefficient of the within the unit variability.

(2) M0:Y(t)=L(t)+σϵ,

(3) L(t)=η(t)+σβB(t),

In addition, Cheng et al. [25] proposed six different models to characterize the degradation path of any materials/devices, with a combination of different setup parameters the six models are presented in Table 3 (Y is indicating if the parameter is included in the model). This approach can be solved by using the package iDemo within R software.

Accelerated destructive degradation test (ADDT) was the second approach used in this paper and it was proposed by Meeker et al, [12]. In an ADDT type of test, the measurements of the response are destructive, where the test specimens are destroyed in order to estimate yield points. A well-known characteristic of accelerated testing is the necessity of extrapolating data, and the experiment is usually carried out under accelerated conditions; however, the model is to be fitted to provide information for real or natural conditions. The destructive degradation model can be expressed as follows (in Equations (4) and (5)):

(4) g(Y)∼F(μ,σ)

(5) μ=h(f(Time),X)

where: *g*(*Y*) is the transformed *Y* variable; *F* is the selected probability distribution; μ is the location parameter, defined by *h*; *h* is a function that relates the transformed time variable and the explanatory variable *X*; σ is the scale parameter of the distribution; *f*(*Time*) is the transformed time variable; *X* is an optional explanatory variable.

The Jmp™ software (by SAS Company) includes a procedure to solve problems for accelerated degradation testing using Escobar and Meeker’s approach. It allows the user to analyze data for any number of variables and to select the best degradation model for the response variable. One particular characteristic is that different combinations of statistical transformations such as linear, logarithmic, and square can be selected for the response. Another characteristic is that a series of probability distribution analysis are considered. The type of distribution used in reliability analysis depends on the behavior of the failure rates. In this paper, a wider range of distributions was analyzed for the failure rates of the material. The software Jmp™ created a linear path of the degradation for each combination transformation/probability distribution; this tool is essential if data are uncertain to follow a normal distribution; in that case, Weibull, log-normal, quadratic, or another distribution could be a better fit. The analysis started with the selection of the transformation and probability distributions for the response. For this analysis, all combinations of the transformation/probability distribution were computed one by one and a summary for the tensile response is presented in Table 4.

### Degradation Models

Table 3, as shown in Section 3, presents different degradation models, the transformation applied to the response, the probability distribution that the response was adjusted to, and the statistical model fit indicators Akaike’s Information Criterion (AIC), Bayesian Information Criterion (BIC), and likelihood. The model with the smallest AIC and BIC was selected as the best fit for the tensile response. The model that best fit the data was the quadratic transformation of the response and a logistic probability distribution, marked in bold in Table 4. The Akaike value was 356.3 which was the smallest amongst the list of the models. This model was selected and further analysis is presented. The model parameters were calculated for the tensile response and presented in Equation (6).

(6) Sqrt(Tensile)=9.303−0.000375(t)+0.2484

**Figure 2:**
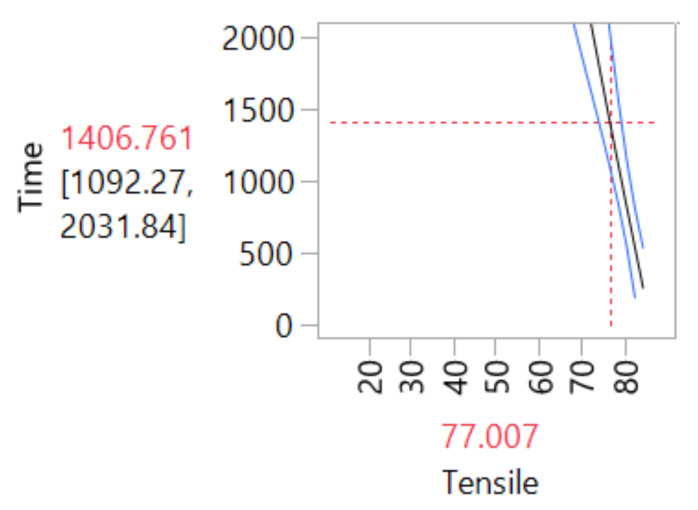
Intersection point in time and 10% of degradation.

The degradation path estimation for tensile response is shown in Figure 2. The 10% value of the deg-radation rate to reach the pseudo failure time was established to test the model. Figure 2 presents the intersection point in time-degradation; when the material would reach this level of degradation with a mean *(µ)* degradation point at 1406.76 h.

**Figure 3:**
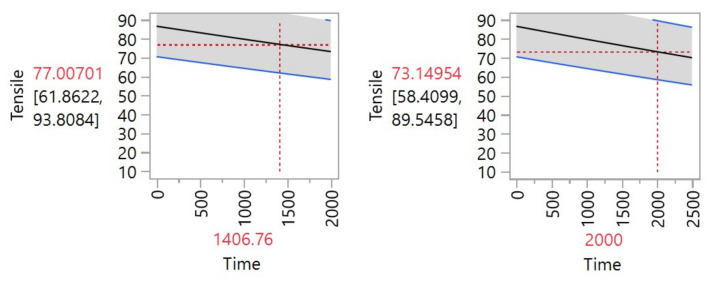
Degradation prediction for tensile (**left**) for 10% and (**right**) for 2000 h.

Figure 3 illustrates what degradation level would be reached at a certain point in time. The graph shows that at 1406 h the tensile strength would be 77.007, which was 10% degradation. Additionally, a predicted degradation rate was calculated for a 2000 h point and the value of the tensile strength at this point was 73.14, which was 15% degradation rate.

The models were analyzed using the Gaussian process and accelerated destructive degradation test to compare both approaches and to select the model that best fits the degradation path of this material. Using AIC, BIC, and likelihood as statistical indicators with an AIC value of 356 (ADDT) compared to a value of 478.076 (Gaussian), it can be concluded that the linear model obtained using accelerated degradation test methodology with jmp™ software was a model with a better fit for the tensile response. The flexural strength results were analyzed with an accelerated destructive degradation test approach using jmp™ software. All combinations of transformation/probability distributions were computed and a summary is presented in Table 5.

The selected model was the one with a quadratic transformation and normal distribution with the lowest AIC, BIC, and log-likelihood values, marked in bold in Table 5. It can be concluded that with an AIC value of 505.8 (ADDT) compared to a 583.9 (Gaussian) the linear model was a better fit for the flexural response. The linear model that represented the degradation´s path for the flexural response would be represented as follows in Equation (7).

(7) Sqrt(Flexural)=11.317−0.003453(t)+2.056

**Figure 4:**
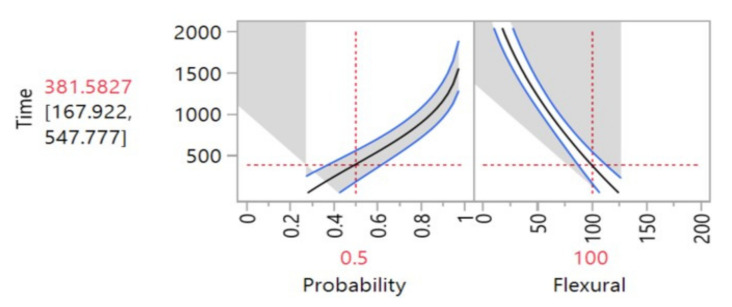
Intersection point in time-degradation: flexural.

Figure 4 presents the estimated degradation path for the selected model. The path was based on a previously established 10% of the flexural strength degradation rate, this 10% degradation rate was to emulate a pseudo fail time. The result is presented and the estimated mean (*µ*) degradation point was at 381.58 h, meaning that the material would be degraded in 10% of its flexural strength after roughly 381 h of exposure.

**Figure 5:**
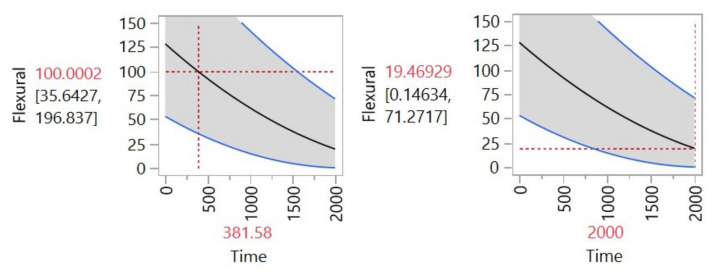
Degradation predictions: flexural.

Figure 5 presents the degradation level that would be reached at a certain point in time, in Figure 5 2000 h was the time selected for the calculation. The graph presents that at 381.58 h the flexural strength would be 100.0002 (10% degradation level). For the 2000 h’ point, the value of the flexural strength was 19.46 (82% degradation rate approximately).

Color change results were analyzed with accelerated destructive degradation test approach using jmp™ software and the Gaussian approach using R software, and the comparison using AICc was of 512.91 (Gaussian) and 320.61 (ADDT), similar to tensile and flexural, ADDT was a better fit for the color response. All combinations of the transformation/probability distribution were computed. The model selected has a quadratic transformation and a normal distribution. With the lowest AIC, BIC, and log-likelihood values. The parameters for the model are computed and presented in Equation (8), this model represents the color´s variable degradation rate of PLA.

(8) Sqrt(Color)=8.6693−0.000411(t)+0.39997

**Figure 6:**
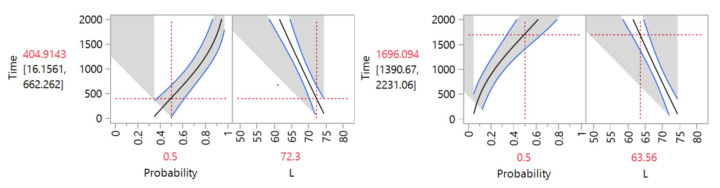
Intersection point in time: (**left**) 10% (**right**) 20% degradation.

**Figure 7:**
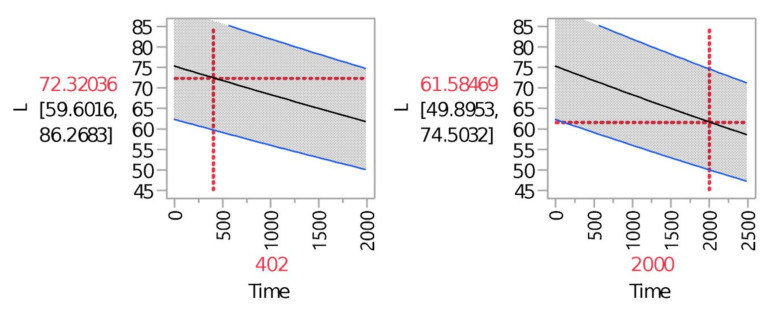
Degradation predictions: color.

Figure 6 and Figure 7 present the degradation path for the model selected for the color variable, which was based on the 10% target established for color degradation rate to reach the pseudo failure time. Figure 6 (left) presents the intersection point in time-degradation when the material reaches this 10% level of degradation with a mean *(µ)* degradation point at 404.094 h. An alternative degradation pseudo failure time for the color variable is presented with an established 20% degradation rate in Figure 6 (right). Results show that the material would be 20% degraded after 1696.09 h.

Figure 7 presents what degradation level will be reached at a certain point in time. The shows that at 402 h the color would be 72.32 (10% degradation). Additionally, a predicted degradation rate was calculated for a 2000 h point and the value of the tensile strength at this point was 61.58 (24% degradation rate).

## 4. Discussion

Hypothesis 1 is the overall hypothesis, and perhaps the most important one in this research. The goal is to identify if the selected response variables were significantly affected by the accelerated degradation variables designated; UV light, humidity, and temperature exposure have a statistical difference in the degradation rate of PLA. The test of this hypothesis was done using the multivariate nonparametric test. Four multivariate tests are included: ANOVA type test, McKeon approximation for the Hotelling Test, Muller approximation for the Bartlett–Nanda–Pillai Test, and Wilks Lambda test; all four tests provided a *p*-value of 0.001. With the small *p*-value, the null hypothesis is rejected. It is concluded that, “there is a statistically significant effect of UV light, humidity, and temperature in the degradation rate PLA after 2000 h. of accelerated weather exposure”. The results indicated that the experimentation was providing useful information to built the models presented in the results section of this work.

This research was focused on the degradation analysis, however, there is a bigger role of manufacturing and engineering involved; the methodology used in this research includes two different manufacturing process: injection and extrusion molding for the fabrication of sample materials. Several responses were selected to evaluate the changes in the material that was exposed to accelerated weathering conditions. Mechanical and physical tests were used to quantify the changes in the material. The changes in the material were analyzed and used to estimate the degradation path of the biopolymer.

The models proposed are intended to be used as a tool for characterization of material in regards to the durability as an engineering material. It is essential to understand material behavior, especially in term of reliability and durability.The results indicate that PLA loses flexural strength faster than tensile strength, this can be attributed to the properties of the material. Based on the literature this biopolymer tends to be brittle. The results of this research quantifies and models this specific characteristic. In terms of physical characteristics, the material loses its lightness sooner that it loses weight, this changes can be attributed to the UV light impact on the material.

Compared with previous studies presented in the introduction, this research include several weathering condition within the experiment in order for the results to be extrapolated to real life conditions. One important output of this research, in comparison with previous models cited in this paper, is that one model was created for each response, tensile, flexural, and changes. Moreover, in comparison with the models presented in Table 1, this research explores in the experimentation the effect of multiple factors accelerating the degradation of the material.

The overall goal of this paper was to present a degradation model that would be able to predict the lifetime of Polylactic acid (PLA); the objectives for this research were met. This paper intended to provide a better understanding of biopolymer degradation. Several responses were selected to evaluate the changes in the material exposed to accelerated weathering conditions. Accelerated degradation destructive testing was successfully applied to the experimentation of the material, and reliable results were obtained after 2000 h of accelerated degradation exposure.

Mechanical and physical tests were used to quantify the changes in the material. The changes in the material were analyzed and used to estimate the degradation path of the biopolymer. By using accelerated destructive testing and Gaussian approaches, a model for each variable was proposed. After analyzing both approaches, the accelerated destructive degradation test approach was a better option than the Gaussian process to model the degradation path of the material in all of the cases studied in this paper.

The research results were successful, multiple degradation models were created. The models can be used to accuratelly predict the lifetime of PLA. The three hypotheses, (1) UV light, humidity, and temperature exposure have a statistical difference in the degradation (2) UV light exposure has a significant effect on PLA degradation rate after an accelerated weather exposure of 2000 h; and (3) acceleration time has effects in the PLA samples, after 250, 500, 1000, and 2000 h of accelerated condition exposure, were tested and each one of them rejected the null hypothesis.

## Figures and Tables

**Table 1:** Existing degradation models.

Model	General Description	Type of Degradation
1. Mechanic or mechanistic models [7].	Using differential equations of the moments, de-polymerization (chemical reaction). They analyzed the degradation in terms of chemical composition and changes in the molecular weight, going from a polymer to a trimer–dimer and finally a monomer.	Thermal
2. Stochastic modeling (simulated information) [8].	They proposed that the lifetime of a system is often related or called as continuous damage increment, assumed as Gaussian processes. An accelerated test must be performed in order to obtain results in a reasonable time frame	Thermal
3. Statistical models (1 variable) [9].	Mass loss was measured as degradation indicator induced by bulk degradation (equally in the material)	Hydrolysis
4. Series of mechanical property comparisons (not a specific model) [10].	Focused their research on poly-caprolactone samples, with applications in tissues engineering area. Extrusion process was performed with the raw material followed by rapid prototyping. Sodium hydroxide was used to accelerate the hydrolysis, during 6 weeks at 37 °C, the samples were monitored every week. Average molecular weight tests were performed and then statistical analysis conducted using Student’s t-test.	Hydrolysis
5. Chemical reaction model [11].	The authors gave a mathematical analysis of the chain-end polymer degradation monomer. The analysis was made with a discrete equation that explained the evolution of the polymer degradation. The authors proposed an alternative “discrete-continuous” in which the discrete part represents the oligomers, whereas the continuous represents the larger polymers.	Not accelerating factor
6. Arrhenius model [12].	Described the temperature influence in degradation. They modeled different temperatures and present the result of the approximation of failure. Temperatures were used from 80 to 237 °C	Thermal
7. Lifetime and probability models (Bayesian approach) [13].	analyzed degradation in complement to a failure time study; they mentioned that degradation is often ignored in these type of models, they only consider the final point of failure and not the influences of the entire degradation process in the material.	Not accelerating factor.
8. Accelerated destructive degradation testing (one variable) [14].	They presented the statistical model for one variable, in this case the accelerated variable was temperature. They mentioned that degradation rate is always a linear function of time, degradation rate might be increasing and decreasing over the course of the product life. In their simulation they used four levels of temperature as degradation factor through five different times going from 0 up to 60 weeks	Thermal

**Table 2:** Experimental sample distribution.

Time	Color	Flexural	Tensile
0/control	C0	F0	T0
250	C1	F1	T1
500	C2	F2	T2
1000	C3	F3	T3
1500	C4	F4	T4
2000	C5	F5	T5
2000 RH (90%)	C6	F6	T6

**Table 3:** Degradation models iDemo.

Model		Variation Source			
		η	ση	σβ	σϵ
1	μ0	Y	Y	Y	Y
2	μ1	Y	Y		Y
3	μ2	Y		Y	
4	μ3	Y	Y	Y	
5	μ4	Y			Y
6	μ5	Y		Y	Y

**Table 4:** Tensile model list.

Transformation	Distribution	Path Definition	Log Likelihood	AIC’c	BIC
Linear	Normal	μ = b0 + b1 * time	224.25	454.93	460.79
Log	Normal	μ = b0 + b1 * time	263.95	534.32	540.17
Quadratic	Normal	μ = b0 + b1 * time	199.54	405.51	411.37
Linear	Logistic	μ = b0 + b1 * time	207.13	420.68	426.54
Log	Logistic	μ = b0 + b1 * time	230.14	466.70	472.56
**Quadratic**	**Logistic**	μ = b0 + b1 * time	**174.98**	**356.38**	**362.23**
Linear	Lognormal	μ = b0 + b1 * time	263.95	534.32	540.17
Log	Lognormal	μ = b0 + b1 * time	279.12	564.66	570.52
Quadratic	Lognormal	μ = b0 + b1 * time	263.95	534.32	540.17
Linear	Log logistic	μ = b0 + b1 * time	230.14	466.70	472.56
Log	Loglogistic	μ = b0 + b1 * time	239.71	485.85	491.70
Sqrt	Loglogistic	μ = b0 + b1 * time	230.14	466.70	472.56
Linear	Weibull	μ = b0 + b1 * time	215.66	437.74	443.60
Log	Weibull	μ = b0 + b1 * time	227.38	461.19	467.05
Sqrt	Weibull	μ = b0 + b1 * time	230.04	466.50	472.35

**Table 5:** Flexural model list.

Transformation	Distribution	Path Definition	Log Likelihood	AIC’c	BIC
Linear	Normal	μ = b0 + b1 * time	294.31	595.05	600.90
Log	Normal	μ = b0 + b1 * time	297.30	601.03	606.89
**Sqrt**	**Normal**	μ = b0 + b1 * time	**249.72**	**505.86**	**511.72**

## Data Availability

The data that support the findings of this study are available from the corresponding author upon request.

## References

[1] Gupta A.P., Kumar V. (2007). New emerging trends in synthetic biodegradable polymers—Polylactide: A critique. Eur. Polym. J..

[2] Jamshidian M., Tehrany E., Imran M., Jacquot M., Desobry S. (2010). Poly-Lactic Acid: Production, Applications, Nanocomposites, and Release Studies. Compr. Rev. Food Sci. Food Saf..

[3] Harris A.M. (2008). Improving mechanical performance of injection molded PLA by controlling crystallinity. J. Appl. Polym. Sci..

[4] Farah S., Anderson D.G., Langer R. (2016). Physical and mechanical properties of PLA, and their functions in widespread applications. Adv. Drug Deliv. Rev..

[5] Matuana L., Jin S., Stark N. (2011). Ultraviolet weathering of HDPE/wood-flour composites coextruded with a clear HDPE cap layer. Polym. Degrad. Stab..

[6] Muller R.-J. (2011). Biodegradability of Polymers: Regulations and Methods for Testing. Biodegradability of Polymers.

[7] Kruse T.M., Woo O.S., Wong H.W., Khan S.S., Broadbelt L.J. (2002). Mechanistic modeling of polymer degradation: A comprehensive study of polystyrene. Macromolecules.

[8] Padgett W.J., Tomilson M.A. (2004). Inference from Accelerated Degradation and Failure Data Based on Gaussian Process Models. Lifetime Data Anal..

[9] Metters A.T., Bowman C.N., Anseth K.S. (2000). A Statistical Kinetic Model for the Bulk Degradation of PLA-b-PEG-b-PLA Hydrogel Networks. Phys. Chem. B.

[10] Lam C.X., Savalani M.M., Teoh S.-H., Hutmacher D.W. (2008). Dynamics of in vitro polymer degradation of poly caprolactone-based scaffolds: Accelerated versus simulated physiological conditions. Biomed. Mater..

[11] Kostoglou M. (2000). Mathematical analysis of polymer degradation with chain-end scission. Chem. Eng. Sci..

[12] Meeker W.Q., Escobar L.A., Lu J. (1998). Accelerated Degradation Tests: Modeling and Analysis. Technometrics.

[13] Pettit L.I., Young K.S. (1999). Bayesian analysis for inverse Gaussian lifetime data with measures of degradation. J. Stat. Comput. Simul..

[14] Shi Y., Escobar L.A., Meeker W.Q. (2009). Accelerated Destructive Degradation Test Planning. Technometrics.

[15] Garlotta D. (2001). A Literature Review of Poly(Lactic Acid). J. Polym. Environ..

[16] Hoshino A., Isono Y. (2002). Degradation of aliphatic polyester films by commercially available lipases with special reference to rapid and complete degradation of poly(L-lactide) film by lipase PL derived from *Alcaligenes* sp. Biodegradation.

[17] Auras R.A., Lim L.T., Selke S.E., Tsuji H. (2011). Poly(Lactic Acid): Synthesis, Structures, Properties, Processing, and Applications.

[18] Fekushima K., Abbate C., Tabuani D., Gennari M., Camino G. (2009). Biodegradation of poly(lactic acid) and its nanocomposites. Polym. Degrad. Stab..

[19] Morrell J., Stark N.M., Pendleton D.E., McDonald A.G. (2006). Durability of Wood-Plastic Composites. Wood Des. Focus.

[20] Kolybaba M.T. Biodegradable Polymers: Past, Present, and Future. Proceedings of the 2003 CSAE/ASAE Annual Intersectional Meeting Sponsored by the Red River Section of ASAE Quality Inn & Suites 301 3rd Avenue North.

[21] Lee H., Lee D.-H. (2019). Prediction of times-to-failure of semiconductor chips using vmin data. Int. J. Ind. Eng. Theory Appl. Pract..

[22] Mun B., Lee C., Jang S.-G., Ryu B., Bae S. (2019). A bayesian approach for predicting functional reliability of one-shot devices. Int. J. Ind. Eng. Theory Appl. Pract..

[23] Escobar L.A., Meeker W.Q. (2006). A review of Accelerated Test Models. Stat. Sci..

[24] Chen Y., Zhou S., Li Q. (2011). Mathematical modeling of degradation for bulk-erosive polymers: Applications in tissue engineering scaffolds and drug delivery systems. Acta Biomater..

[25] Cheng Y.-S., Peng C.-Y. (2012). Integrated Degradation Models in R Using iDEMO. J. Stat. Softw..

